# *In Vitro* Genotoxicity Screening and Lipid Oxidation in Pork and Chicken Burgers: Effect of Cooking and Gastrointestinal Digestion

**DOI:** 10.3390/ijms27093985

**Published:** 2026-04-29

**Authors:** Amaya Azqueta, Iciar Astiasaran, Diana Ansorena

**Affiliations:** 1 Department of Pharmaceutical Sciences, Faculty of Pharmacy and Nutrition, Instituto de Nutrición y Salud, University of Navarra, Irunlarrea 1, 31008 Pamplona, Spain; 2 Department of Nutrition, Food Science and Physiology, Center for Nutrition Research, Faculty of Pharmacy and Nutrition, Instituto de Nutrición y Salud, University of Navarra, Irunlarrea 1, 31008 Pamplona, Spain; iastiasa@unav.es (I.A.); dansorena@unav.es (D.A.); 3 IdiSNA, Navarra Institute for Health Research, 31002 Pamplona, Spain

**Keywords:** meat burgers, *in vitro* digestion lipid oxidation, genotoxicity, SOS/umu test

## Abstract

The intensity of lipid oxidation after cooking and after *in vitro* gastrointestinal digestion of pork and chicken burgers was assessed. Pan frying with olive or sunflower oils and oven treatment were used as cooking technologies in both types of burgers. Thiobarbituric acid reactive substances (TBARs) were measured after cooking and in the micellar (bioaccessible) and residual (pellet) fractions after gastrointestinal *in vitro* digestion. Genotoxicity was assessed in the micellar fraction using the SOS/umu genotoxicity test. Lipid fraction suffered significant oxidation increases in all samples during the digestion process, especially in oven-treated samples. In general, the bioaccessible fraction showed a higher amount of oxidation products than the residual phase. None of the samples showed genotoxicity activity in the SOS/umu test.

## 1. Introduction

Contaminants in food are substances that occur unintentionally, originating from production, processing, environmental factors, or culinary treatments. Among them, process contaminants are formed during thermal treatment and may include compounds associated with genotoxic and carcinogenic effects. Cooking meat at high temperatures is known to generate genotoxic compounds such as heterocyclic aromatic amines (HAAs), polycyclic aromatic hydrocarbons (PAHs) and N-nitroso compounds (NOCs) [1,2,3]. Within this framework, the International Agency for Research on Cancer classified processed meat as carcinogenic to humans (Group 1) and red meat as probably carcinogenic (Group 2A), mainly drawing on epidemiological evidence associating high intake with colorectal and other cancers [3]. Nevertheless, the mechanisms driving these associations are not fully elucidated, although genotoxicity is considered to play a central role.

Meat is a highly complex matrix in which multiple compounds may coexist and interact. Therefore, evaluating meat as a whole mixture rather than assessing isolated compounds provides a more realistic approximation to human exposure. In line with this, the European Food Safety Authority (EFSA) guideline on the “Genotoxicity assessment of chemical mixtures” recommends testing whole mixture when full chemical characterization is not feasible [4]. EFSA and FAO/WHO propose a stepwise testing strategy for genotoxicity assessment, typically starting with a basic *in vitro* battery that includes the bacterial reverse mutation (Ames) test and the *in vitro* micronucleus assay to cover gene mutations and chromosomal damage [5,6].

However, applying this complete strategy to the wide variety of meat samples (differing in animal origin, processing conditions or cooking methods) is extremely demanding in terms of time and resources. This highlights the need for rapid and sensitive screening approaches. In this context, the SOS/umu genotoxicity assay constitutes a useful alternative [7,8]. This bacterial test is based on the induction of the SOS DNA repair response, which enables the detection of DNA-damaging agents in a quantitative and relatively high-throughput manner. Moreover, a high concordance with the Ames test has been reported [8,9], supporting its use as a preliminary screening tool. From a toxicological perspective, assessing the bioaccessible fraction of meat may be more relevant than analyzing total extractable compounds. In recent years, the effect of the digestion process over different compounds present in foods has been analyzed through *in vitro* methods, but mainly for their nutritional assessment. For their safety evaluation, it has to be pointed out that not all genotoxic substances present in cooked meat will necessarily become available for absorption in the gastrointestinal tract. *In vitro* digestion models, simulating oral, gastric and intestinal phases, allow the isolation of the bioaccessible fraction that would be potentially available for systemic exposure. Combining *in vitro* digestion protocols with genotoxicity assays may therefore provide a more physiologically relevant estimation of the actual risk associated with meat consumption.

Gastrointestinal digestion has been reported as a pro-oxidative environment, leading to increased lipid oxidation [10,11,12,13,14]. Among the resulting compounds, wide types of secondary lipid oxidation products formed during this process (ketones, alcohols, furans, alkenes, among others), aldehydes have been better explored due to their reactivity. The bioavailability and safe dose for lipid-oxidation-derived aldehydes remains unknown [14], and the evaluation of the genotoxicity in the bioaccessible fraction whose malondialdehyde (MDA) content is known might contribute to shedding light onto this issue. In addition to MDA, which has been traditionally used as a marker of lipid oxidation in foods and biological systems, others, such as 4-hydroxy-2-nonenal (HNE) and 4-oxo-2-nonenal (4-ONE), may cause DNA and protein damage through adduct covalent binding [15]. Thus, although the genotoxicity of lipid oxidation compounds has been already described [16], this has not been tested in samples subjected to gastrointestinal digestion. To date, only a limited number of studies have evaluated the genotoxic or mutagenic potential of meats following gastrointestinal digestion [17,18].

Thus, the aim of this study was to evaluate lipid oxidation in fried and oven-cooked pork and chicken burgers subjected to *in vitro* gastrointestinal digestion (INFOGEST), and to assess the associated genotoxic potential. MDA was determined as a marker of lipid oxidation, and genotoxicity was evaluated using the SOS/umu assay, in order to explore the relationship between oxidative processes during digestion and the formation of DNA-damaging compounds.

## 2. Results and Discussion

The composition of the cooked pork and chicken burgers is shown in Table 1. In general, the amounts of protein and fat can be considered as representative for this type of products. Grilled pork burgers have shown values of protein around 28.1–31.1% and of fat around 11.8–12.9% [19] and steamed pork burgers reported 29.72% protein and 12.09% fat [20]. In steamed chicken burgers, lower fat content has been observed (4.23%), with similar protein values (28.04%) [20]. Similarly, in this work, a lower fat content was observed in chicken burgers as compared to pork ones, regardless of the cooking technology applied. Also, some statistical differences can be observed in moisture and protein content between oven-treated and fried samples, but were not relevant from a nutritional standpoint.

As a lipid oxidation marker, the MDA values of cooked and digested samples are reported in Table 2. Cooked samples showed values within the range previously observed for meat dishes (0.40–4.65 mg MDA/kg sample) [21]. Chicken samples, for all cooking treatments, gave rise to higher MDA values as compared to pork burgers. Despite their lower fat content, the higher unsaturation degree of chicken fat as compared to pork fat [22] might explain the higher oxidation susceptibility in these samples.

Digestion, as expected, enhanced the lipid oxidation intensity in all samples, with MDA increment values in the bioaccessible fraction, as compared to cooked samples, ranging from 220–290% for fried samples to 400–480% in oven-treated samples. Oven treatment reported the highest oxidation rates (*p* < 0.001) in this micellar fraction for both species. In a previous work, MDA increments between 31 and 610% were reported in pork-based meat products, with notable differences among them depending on the culinary treatment and ingredients included [21]. In contrast to cooked samples, no significant effect was noticed between species for any cooking treatment. Regarding the pellet, significant increments were also observed as compared to cooked samples, with higher values in pork than in chicken samples, and with frying than in oven-treated samples.

Genotoxicity was assessed in the micellar phase using the SOS/umu test. Although it would also have been interesting to evaluate the genotoxicity of the total digestion or the pellet, the nature of these types of samples makes it difficult to perform a direct *in vitro* analysis without applying extraction methods, and, thus, without analyzing the whole matrix. Moreover, the micellar phase is the fraction that is expected to be absorbed, and therefore the focus of the study is highly relevant.

**Figure 1:**
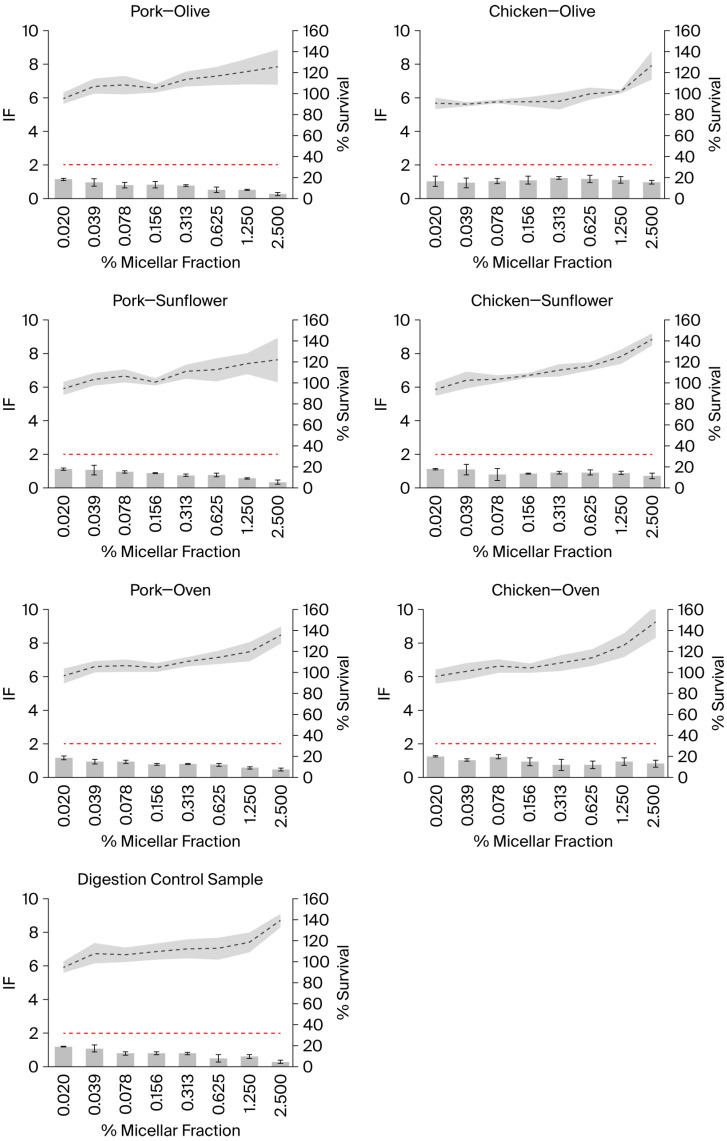
Genotoxicity of the micellar phase of different meat samples prepared using different cooking methods and digested *in vitro*, as well as of the digestion control sample, assessed using the SOS/umu test without external metabolic activation. Data were obtained from three independent experiments. The dotted grey line represents the mean % survival, while the surrounding grey area represents the SD. Grey bars represent the mean induction factor (IF), and their error bars indicate the SD. The red line indicates an induction factor (IF) of 2; a compound is considered genotoxic when the induction factor (IF) is ≥2 at non-toxic concentrations (i.e., % survival > 80%) for the bacteria under any of the tested conditions. Data with % survival higher than 110% were not included in the interpretation of the results.

**Figure 2:**
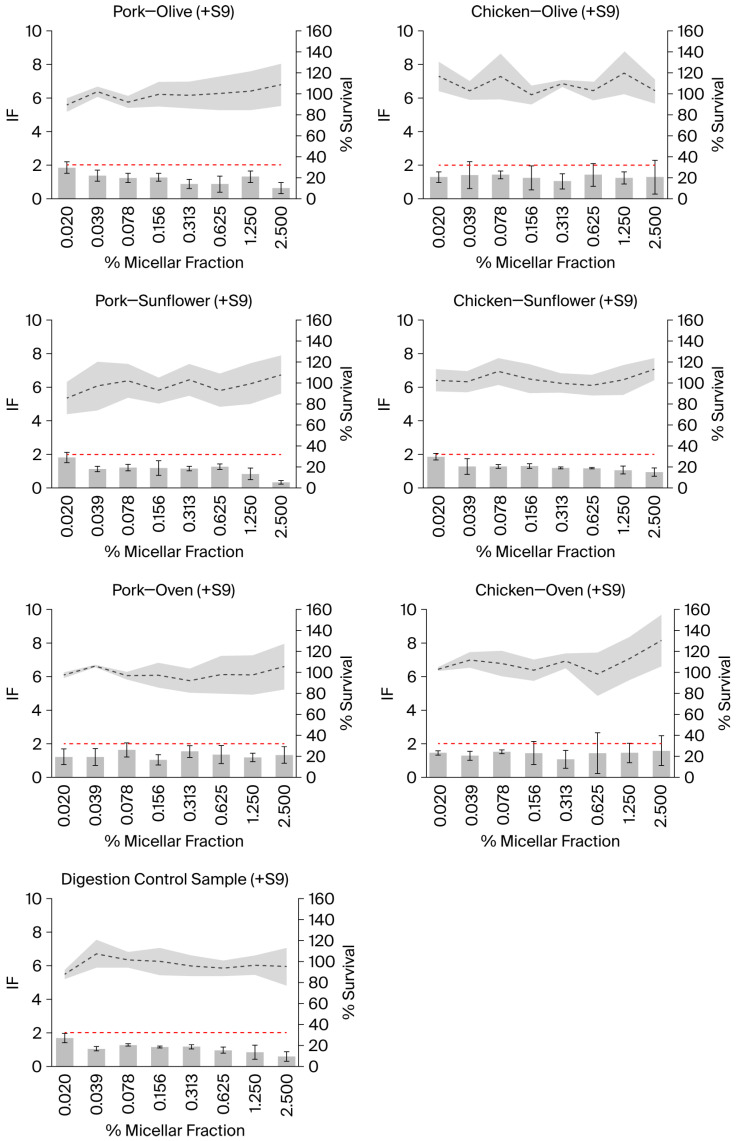
Genotoxicity of the micellar phase of different meat samples prepared using different cooking methods and digested *in vitro*, as well as of the digestion control sample, assessed using the SOS/umu test with external metabolic activation (+S9). Data were obtained from three independent experiments. The dotted grey line represents the mean % survival, while the surrounding grey area represents the SD. Grey bars represent the mean induction factor (IF), and their error bars indicate the SD. The red line indicates an induction factor (IF) of 2; a compound is considered genotoxic when the induction factor (IF) is ≥2 at non-toxic concentrations (i.e., % survival > 80%) for the bacteria under any of the tested conditions. Data with % survival higher than 110% were not included in the interpretation of the results.

None of the tested samples showed a positive response, nor did the digestion control sample, either in the presence or absence of metabolic activation (Figure 1 and Figure 2). The positive and negative controls showed the expected response. No toxicity (i.e., % survival < 80%) was observed in any of the tested samples.

In samples tested in the absence of external metabolic activation, an increase in the % survival was observed as the concentration of the samples increased, exceeding 100% at the highest concentrations (Figure 1). This effect was also observed in the digestion control samples, which could suggest that compounds present in the digestion control samples reach absorbance at 600 nm. However, the analysis of the absorbance of the samples at 600 nm, including the digestion control sample, in the absence of bacteria (but including the bacterial medium), did not show an increase. Therefore, it appears that the increase in the absorbance may result from an interaction between the samples and the bacteria, or that the samples (including the digestion control samples) are able to induce bacterial overgrowth, a possibility that requires further investigation. In any case, the conditions in which the mean % of survival was higher than 110% were not considered for the interpretation of the results; however, their inclusion would not have altered the results. The same criterion was applied to the results of the experiments performed in the presence of external metabolic activation, although, in this case, a % of survival higher than 100% was observed in some samples and not depending on the concentration (Figure 2). Moreover, it was not observed in the digestion control sample. However, one of the advantages of the SOS/umu test is that eight concentrations are tested in each experiment instead of the three or five concentrations that are normally tested in other genotoxicity assays; this gives the researcher the opportunity not to consider the samples in which precipitation or color interference is observed and still have a good number of concentrations tested.

In this context, it is important to consider that the genotoxic potential of the well-characterized lipid-peroxidation-derived aldehyde HNE has been demonstrated in SOS/umu assays, where it acts as a potent inducer of the SOS response in the absence of metabolic activation [23]. Similarly, 4-ONE, although no data are currently available in SOS/umu assays to our knowledge, is a highly electrophilic lipid-peroxidation-derived aldehyde that forms reactive etheno-DNA adducts, supporting its potential to induce DNA damage [24].

It is worth mentioning that the micellar fraction had an oily consistency; however, this was lost after the serial dilutions in DMSO. Moreover, to ensure adequate bacterial exposure, the samples were subjected to orbital shaking during the incubation period, which is part of the standard SOS/umu test procedure.

Numerous studies have investigated the genotoxic potential of extracts obtained from fried meat. A review on this subject reported that extracts from fried meat frequently induced positive responses in *S. typhimurium* TA98 strains when metabolic activation (S9) is included [25]. While the majority of the studies focused on beef and pork, extracts derived from other types of meats, such as mutton, horse, goat, chicken, and lamb, have also demonstrated mutagenic activity in the *S. typhimurium* TA98/TA1538 reversion assay when S9 is used. Only a limited number of studies have employed assays other than the Ames test. A year later, the same authors found no effect of 10 extracts of fried meat-based food from different catering companies in the miniaturized Ames test, but five of them induced chromosomal aberrations after 24 h treatment of TK6 cell [26].

However, the genotoxicity of meat samples after gastrointestinal digestion, which is more relevant to human exposure, has been only marginally studied. Kim et al. evaluated the mutagenicity of pork batter subjected to simulated gastrointestinal digestion using the Ames test with *Salmonella typhimurium* strains TA98 and TA100 and external metabolic activation, thus analyzing only the effect of metabolically activated mutagenic compounds [17]. The *in vitro* digestion model used included enterobacteria (*Escherichia coli* and/or *Lactobacillus sakei*) to simulate intestinal conditions and to assess their influence on mutagenicity. The study also examined the effects of hemin addition and cooking temperature, reporting that both factors increased mutagenicity, whereas mutagenicity decreased during the digestion process, particularly during the intestinal stage in the presence of enterobacteria. However, mutagenicity was reported as the number of revertants per plate, and the comparison with spontaneous revertant levels was not explicitly presented, making it difficult to assess whether the observed responses fulfilled the classical Ames test criteria for mutagenicity.

The genotoxicity of cured meat (salami) after *in vitro* gastrointestinal digestion (without including the colonic fermentation), comparing different formulations designed to reduce or replace nitrate/nitrite additives, has been evaluated [18]. Digested salami samples were applied to HT-29 human colon cells for 24 h, and genotoxicity was evaluated using the standard comet assay. The results showed that none of the digested salami formulations induced DNA damage compared with untreated control cells, although the digests exhibited concentration-dependent antiproliferative effects. Unfortunately, none of the previously mentioned papers that assessed genotoxicity in meat and digested meat reported lipid oxidation values to be compared to our results.

In summary, despite the increase in lipid oxidation markers, none of the bioaccessible fractions induced genotoxic effects in the SOS/umu assay, either in the absence or presence of external metabolic activation, at the conditions tested. These results suggest that the potentially absorbable oxidation products generated during the cooking and gastrointestinal digestion of pork and chicken burgers do not appear to produce a genotoxic effect detectable by this screening assay.

Overall, the combination of *in vitro* gastrointestinal digestion with rapid genotoxicity screening tests represents a useful approach for evaluating the potential biological effects of complex and highly diverse food matrices. This strategy may contribute to a more realistic assessment of the safety of cooked meat products by focusing on the fraction that becomes bioaccessible during digestion.

## 3. Material and Methods

### 3.1. Burgers Elaboration

#### Formulation and Preparation of Burgers

Ground pork (head end of loin, lean) and chicken meat (leg, skinless) (3 kgk each) were purchased in a local market on three different days to obtain different batches and ensure the representativeness of the samples. Every meat batch was salt added (1%) and conveniently homogenized. No other ingredients were added.

A total of 18 burgers (80 g/burger) for each type of meat were prepared using a burger mold. Six of these burgers were cooked in an oven at 180 °C for 24 min (12 min each side). The other 12 burgers were divided into two groups, for being fried with two different oils: olive and sunflower. In both cases, burgers were fried with 5 mL of oil and 3 min each side, until reaching an internal temperature of 75 °C. Cooked samples were vacuum packed and kept at -20 °C until analysis.

### 3.2. Proximate Composition

Moisture, ash and protein content were measured in cooked beef and chicken burgers using the official methods 950.46, 920.153, and 928.08 [27,28,29]. Fat content was determined using a Soxhlet extractor B-811 Büchi Extraction System (Flawil, Switzerland) [30]. Every parameter was measured in quadruplicate (analytical replicates).

### 3.3. In Vitro Digestion

The *in vitro* digestion model consisted of three sequential phases (oral, gastric and intestinal digestion), following the standardized procedure described in the INFOGEST protocol [31]. For the oral phase, 5 g of sample was mixed with 4.5 mL of pre-heated SSF (simulated salivary fluid). Then, 25 µL of 0.3M CaCl_2_(H_2_O)_2_ and α-amylase (providing a final activity of 75 U/mL in the final digestion mixture) were added and the sample was incubated at 37 °C for 2 min. For the gastric phase, 8 mL of SGF (simulated gastric fluid), 5 µL of 0,3 M CaCl_2_(H_2_O)_2_, pepsine (equivalent 2000 U/mL final digestion volume) and gastric lipase (final activity of 60 U/mL final digestion volume) were added. The pH was adjusted to 3 with 5 M HCl and the mixture was incubated for 2 h at 37 °C. Finally, to simulate intestinal digestion, 8.5 mL of SIF (simulated intestinal fluid), 40 µL of 0.3 M CaCl_2_(H_2_O)_2_, and pancreatine and bile salts (equivalent 100 U/mL of trypsin and 10 nM of bile salts) were added, the pH was adjusted to 7 with 1 M NaOH, and the sample was incubated for 2 h at 37 °C. Then, after the digestion process, samples were centrifuged at 2000× *g*, at 4 °C for 50 min (Eppendorf centrifuge 5810R) to separate the bioaccessible phase (micellar phase) from the residual fraction (pellet) [32]. Three digestions per type of cooked sample and a control digestion (sample replaced by water) were performed. Fractions were kept frozen at −20 °C until analysis.

### 3.4. Lipid Oxidation

Lipid oxidation in cooked and digested samples was determined using the TBARs method following the protocol described elsewhere [11]. Briefly, 150 mg (for cooked samples) or 400 mg (for the micellar fraction and pellet) were mixed with 7.5% trichloroacetic acid (TCA) to a final volume of 1 mL. Subsequently, 40 µL of 4.5% (*w*/*v*) butylated hydroxytoluene (BHT) in ethanol was added. Samples were centrifuged (5 min at 2200× *g*), and 750 µL of the supernatant was reserved. The residue was combined with 250 µL TCA 7.5%. After a second centrifugation, 250 µL of the supernatant was pooled with the 750 µL previously obtained. An aliquot (250 µL) was reacted with an equal volume of 40 mM TBA solution prepared in acetic acid. The reaction mixtures were heated at 90 °C for 45 min and then cooled in ice for 10 min. Absorbance was measured at 532 nm in microplates using 1,1,3,3-tetraethoxypropane (TEP) as a standard, and results were expressed as mg MDA/kg sample. All analyses were conducted in triplicate.

### 3.5. SOS/umu

The SOS/umu test was carried out following previously reported protocols [7,8] with minor adaptations. The experiments were conducted both in the absence and in the presence of an external metabolic activation system consisting of 10% rat S9 mix (prepared from Aroclor-induced SD rat liver S9 fraction; Trinova, Giessen, Germany). The bacterial strain used was Salmonella typhimurium 1535/pSK1002, obtained from the German Collection of Microorganisms and Cell Cultures (DSMZ), and preserved at −135 °C in TGA medium supplemented with 10% DMSO. After thawing, 0.5 mL of the bacterial stock was inoculated into 100 mL TGA medium containing 50 µg/mL ampicillin, taking advantage of the strain’s ampicillin resistance for selective growth. Cultures were incubated at 37 °C under gentle orbital agitation until reaching an optical density at 600 nm (OD600) between 0.5 and 1.5 (approximately 15–17 h). Subsequently, the culture was diluted in TGA medium without ampicillin and further incubated for 2 h at 37 °C with mild shaking until obtaining the bacterial culture in exponential growth phase (OD600 between 0.15 to 0.4).

For the micellar fraction treatment, samples were thawed and subjected to 11 consecutive 1/2 serial dilutions in DMSO using a 96-well U-bottom plate (plate A), with a final volume of 10 µL per well. A digestion control (i.e., sample from an *in vitro* digestion performed without meat) was included. Negative and positive controls were also incorporated: DMSO as negative control, while 4-nitroquinoline-n-oxide (4-NQO, Sigma-Aldrich, St. Louis, MO, EEUU; maximum concentration tested 2.5 µg/mL) and 2-aminoanthracene (2-AA, Sigma-Aldrich, St. Louis, MO, EEUU: maximum concentration tested 12.5 µg/mL) were used as positive controls in the absence and presence of S9 mix, respectively. Subsequently, 70 µL of water were added to each well.

Two additional 96-well plates were then prepared (plates B): one plate received 10 µL of S9 mix per well, whereas the other received 10 µL of PBS. Afterwards, 25 µL from plate A and 90 µL bacterial suspension in exponential phase were added to each well of both plates. The plates were incubated for 4 h at 37 °C with orbital shaking.

Following incubation, absorbance at 600 nm was measured in plates B to assess toxicity. Bacterial survival (%) was calculated as the ratio between the A600 value of each tested concentration and the mean A600 of the negative control, multiplied by 100.

To evaluate the induction of the SOS response, β-galactosidase activity was determined. For this purpose, 30 µL from plates B was transferred to new 96-well plates containing 150 µL per well of 0.9 mg/mL 2-nitrophenyl-β-D-galactopyranoside (ONPG, Sigma-Aldrich, St. Louis, MO, EEUU) prepared in B-buffer. These plates (plates C) were incubated for 30 min at 28 °C under orbital shaking and protected from light. The enzyme reaction was stopped by adding 120 µL of 1M Na_2_CO_3_ per well.

To determine β-galactosidase activity in relative units (RU), the absorbance of plates C were measured at 420 nm and these values were divided by the absorbance measured at 600 nm (to evaluate the toxicity). Finally, the induction factor (IF) was calculated by dividing the RU of each sample by the RU of the negative controls (calculated using the average absorbance at 600 and 420 nm).

A sample is considered to be genotoxic when the IF is ≥2. Three independent experiments were performed.

### 3.6. Statistical Analysis

General composition and lipid oxidation data are expressed as mean ± standard deviation (SD). A two-way analysis of variance (ANOVA) was applied to assess the effects of the two independent factors (*Species and Cooking*) as well as their interaction on the dependent variables. When a significant interaction was observed, the effects of each factor were further analyzed using one-way ANOVA, followed by Bonferroni’s post hoc tests for multiple comparisons. Statistical significance was established at *p* < 0.05.

## Figures and Tables

**Table 1:** General composition data on the six types of cooked burgers. Data are expressed on g/100g burger.

	Olive			Sunflower			Oven					
	Pork	Chicken	*p*	Pork	Chicken	*p*	Pork	Chicken	*p*	P Species	P Cooking	P Interaction
**Moisture**	58 ± 1	58 ± 1		59.26 ± 0.66	58 ± 1		51 ± 1	57 ± 2		NS	**	NS
**Protein**	26.21 ± 0.20 b	27.10 ± 0.98	NS	24.12 ± 0.12 a	27.75 ± 0.72	***	32 ± 1 c	29 ± 1	NS	NS	***	***
**Fat**	15 ± 2 ab	11.71 ± 0.45	*	15.54 ± 0.60 b	11 ± 1	***	12.50 ± 0.73 a	10.77 ± 0.21	*	***	*	*
**Ash**	1.39 ± 0.18	1.13 ± 0.08		1.46 ± 0.20	1.10 ± 0.02		1.16 ± 0.14	1.17 ± 0.11		**	NS	NS

A two-way ANOVA was applied to analyze the effects of the two factors, *Species* and *Cooking* (NS: *p* > 0.05; *: *p* < 0.05; **: *p* < 0.01; ***: *p* < 0.001). When a significant interaction was detected (*p* < 0.05), one-way ANOVA followed by Bonferroni’s *post hoc* test was performed to identify differences among samples within each factor. For each parameter, different lowercase letters indicate significant differences among cooking conditions within each species (*p* < 0.05), and *p* values are reported for comparisons between species within each cooking condition.

**Table 2:** Lipid oxidation data of the six types of burgers. TBAs (mg MDA/kg sample).

	Olive			Sunflower			Oven					
	Pork	Chicken	*p*	Pork	Chicken	*p*	Pork	Chicken	*p*	P Species	P Cooking	P Interaction
**TBA—Cooked**	1.11 ± 0.09 aA	1.24 ± 0.04 aA	**	1.27 ± 0.02 bA	1.34 ± 0.04 bA	***	1.17 ± 0.06 aA	1.41 ± 0.06 bA	***	***	***	***
**TBA—Micellar phase**	3.80 ± 0.26 B	3.56 ± 0.05 C		3.28 ± 0.12 B	3.64 ± 0.12 C		5.57 ± 0.04 C	6 ± 1 C		NS	***	NS
**TBA—Pellet**	4.80 ± 0.06 abC	2.88 ± 0.02 bB	***	5.98 ± 0.10 bC	2.97 ± 0.07 bB	***	3.57 ± 0.85 a B	1.89 ± 0.09 aB	*	***	***	***

A one-way ANOVA followed by Bonferroni’s post hoc test was used to evaluate differences among the three different conditions (cooked samples, micellar fraction and pellet) for each type of burger. Different capital letters in the same column indicate significant differences (*p* < 0.05). A two-way ANOVA was applied to analyze the effects of the two factors, *Species* and *Cooking* (NS: *p* > 0.05; *: *p* < 0.05; **: *p* < 0.01; ***: *p* < 0.001). When a significant interaction was detected (*p* < 0.05), one-way ANOVA followed by Bonferroni’s *post hoc* test was performed to identify differences among samples within each factor. Different lowercase letters indicate significant differences among cooking conditions within each species (*p* < 0.05), and *p* values are reported for comparisons between species within each cooking condition.

## Data Availability

The original contributions presented in this study are included in the article. Further inquiries can be directed to the corresponding author.
